# Spatiotemporal metabolomics of tobacco leaves in response to *Pseudomonas syringae* infection

**DOI:** 10.1093/plphys/kiag623

**Published:** 2026-08-20

**Authors:** Xinhua Tian, Zechao Qu, Jiaqi Wang, Lijun Meng, Huan Su, Qian Sun, Jiemeng Tao, Peng Lu, Qiao Wang, He Li, Jianfeng Zhang, Peijian Cao, Yuanhu Xuan, Jingjing Jin

**Affiliations:** China Tobacco Gene Research Center, Zhengzhou Tobacco Research Institute of CNTC, Zhengzhou 450001, China; Beijing Life Science Academy, Beijing 102200, China; Department of Plant Pathology, College of Plant Protection, Shenyang Agricultural University, No. 120 Dongling Road, Shenhe District, Shenyang, Liaoning 110866, China; China Tobacco Gene Research Center, Zhengzhou Tobacco Research Institute of CNTC, Zhengzhou 450001, China; China Tobacco Gene Research Center, Zhengzhou Tobacco Research Institute of CNTC, Zhengzhou 450001, China; Beijing Life Science Academy, Beijing 102200, China; China Tobacco Gene Research Center, Zhengzhou Tobacco Research Institute of CNTC, Zhengzhou 450001, China; China Tobacco Gene Research Center, Zhengzhou Tobacco Research Institute of CNTC, Zhengzhou 450001, China; Beijing Life Science Academy, Beijing 102200, China; Department of Plant Pathology, College of Plant Protection, Shenyang Agricultural University, No. 120 Dongling Road, Shenhe District, Shenyang, Liaoning 110866, China; China Tobacco Gene Research Center, Zhengzhou Tobacco Research Institute of CNTC, Zhengzhou 450001, China; Beijing Life Science Academy, Beijing 102200, China; China Tobacco Gene Research Center, Zhengzhou Tobacco Research Institute of CNTC, Zhengzhou 450001, China; Beijing Life Science Academy, Beijing 102200, China; China Tobacco Gene Research Center, Zhengzhou Tobacco Research Institute of CNTC, Zhengzhou 450001, China; Beijing Life Science Academy, Beijing 102200, China; China Tobacco Gene Research Center, Zhengzhou Tobacco Research Institute of CNTC, Zhengzhou 450001, China; China Tobacco Gene Research Center, Zhengzhou Tobacco Research Institute of CNTC, Zhengzhou 450001, China; Beijing Life Science Academy, Beijing 102200, China; China Tobacco Gene Research Center, Zhengzhou Tobacco Research Institute of CNTC, Zhengzhou 450001, China; Beijing Life Science Academy, Beijing 102200, China; State Key Laboratory of Elemento-Organic Chemistry and Department of Plant Protection, National Pesticide Engineering Research Center, Nankai University, 94 Weijin Road, Tianjin 300071, China; China Tobacco Gene Research Center, Zhengzhou Tobacco Research Institute of CNTC, Zhengzhou 450001, China; Beijing Life Science Academy, Beijing 102200, China

## Abstract

Tobacco (*Nicotiana tabacum* L.) is a major economic crop and a model for plant–pathogen interactions, yet the spatiotemporal dynamics of defense metabolism during infection remain poorly characterized. Here, we used MALDI-MSI-based spatial metabolomics to systematically profile tobacco leaves during *Pseudomonas syringae* infection. Multidimensional analysis of 1,399 annotated metabolites revealed distinct spatiotemporal regulation patterns. Temporally, early infection (12 h postinfection (hpi)) was characterized by increased organic acids and terpenoids, followed by a mid-stage shift toward phenolic acids and quinones (24 hpi) and a late-stage enrichment of alkaloids by 60 hpi. Spatially, constrained clustering produced anatomy-aligned segmentation maps and revealed cell type preferences across epidermal, mesophyll, and vascular regions, with directional redistribution of differentially expressed metabolites as infection progressed. Defense hormones, including salicylic acid (SA) and jasmonic acid (JA), preferentially accumulated in vascular bundles and varied dynamically over time. Functional validation through exogenous application of representative metabolites (eg calystegine C1 and L-phenylalanine) and hormones (JA and SA), together with genetic manipulation of JA-biosynthetic genes, confirmed their roles in reducing lesion development and suppressing bacterial proliferation. Notably, epidermal enrichment of alkaloids—especially nicotine—and amino acid derivatives showed a decrease-then-increase pattern consistent with early consumption and later replenishment; nicotine's defensive contribution was further supported using a low-nicotine mutant. Collectively, *P. syringae* infection orchestrates a coordinated, cell type-compartmentalized defense metabolic program in tobacco, providing a resource for mechanistic studies and metabolic engineering of disease resistance. This time- and tissue-resolved atlas links metabolite remodeling to hormone-associated signaling and chemical barrier formation during wildfire disease progression.

## Introduction

Tobacco (*Nicotiana tabacum* L.) is a globally significant economic crop and a crucial model plant for studying plant–pathogen interactions. Its productivity is influenced by various biotic and abiotic stresses (Xiang et al. 2022). *Pseudomonas syringae*, as 1 of the top 10 plant pathogens, infects through wounds or stomata, causing diverse foliar diseases and threatening a wide range of economically important crops (Luo et al. 2019). Wildfire disease, a particularly devastating threat to tobacco, is also caused by *P. syringae* (Xin et al. 2018). Toxins, such as tabtoxin secreted by the pathogen, can disrupt mesophyll cells, leading to characteristic chlorotic and necrotic lesions, premature senescence, defoliation, and reduced leaf quality (Cameron and Sarojini 2014). Wildfire outbreaks can reduce tobacco yields by 15% to 20% and cause complete crop failure when it is severe, resulting in hundreds of millions of dollars of annual economic losses (Liu et al. 2021). Breeding disease-resistant cultivars is a common strategy for managing *P. syringae* infections. However, the development of these cultivars often depends on limited genetic resources, such as wild varieties or intraspecific hybridization, leading to a narrow genetic background and limited specificity (Abdellatif et al. 2020; Wang et al. 2023). As 1 of the most widely cultivated nonfood crops, tobacco supports substantial agricultural economies in many countries and serves as a classical experimental system in plant molecular biology due to its well-characterized genome and amenability to genetic manipulation. Consequently, elucidating the mechanisms underlying tobacco–pathogen interactions has both direct agronomic relevance and broader significance for understanding disease resistance across the broader Solanaceae family and beyond.

Driven by advances in molecular biology techniques and molecular breeding research, metabolomics has become crucial for understanding plant disease resistance mechanisms. By analyzing the correlation between metabolites and disease resistance, a growing body of research is elucidating plant molecular responses to pathogens. For instance, metabolomic analyses in resistant and susceptible tomato varieties have demonstrated the dynamic accumulation of key defense metabolites, such as salicylic acid (SA), and have systematically elucidated the disease resistance pathways driven by metabolic reprogramming (Liu et al. 2023). In tobacco, time-series transcriptome and metabolome profiling during *P. syringae* infection revealed *WRKY6*/*WRKY23*-associated metabolic reprogramming that modulated L-phenylalanine and xanthosine branches, highlighting these metabolites as key components linked to disease outcome (Tian et al. 2026). Additionally, research on downy mildew-resistant melons has identified metabolic reprogramming features involving flavonoid inhibition and lignin synthesis, clarifying the collaborative role of the phenylpropanoid pathway and gene regulatory networks in disease resistance (Ling et al. 2023). Studies on cabbage resistance to black rot have also revealed the activation of the SA pathway and the direct antimicrobial effects of flavonoid metabolites, such as chlorogenic acid and caffeic acid (Sun et al. 2022). Precise identification of key metabolites provides novel biomarkers and theoretical frameworks for marker-assisted breeding, making metabolomics an important dimension in modern crop resistance improvement. A wide array of plant metabolomics techniques, including gas chromatography (GC), liquid chromatography (LC), capillary electrophoresis (CE), and hyphenated techniques like GC-/LC-/CE-mass spectrometry (MS) and nuclear magnetic resonance (NMR), are employed for comprehensive analysis of plant metabolites (van Beek 2002). Despite their effectiveness in identifying a broad range of metabolites, these methods typically rely on tissue homogenates, thus lacking information about the spatial distribution of metabolites within the plant. The complex organization of plants, with their diverse array of tissue and cell types, underscores the importance of understanding metabolite spatial localization (Goldberg 1988). Specific metabolites often exhibit asymmetric distributions across different cell types and subcellular compartments within these tissues. Consequently, elucidating the spatial distribution of metabolites is critical for a comprehensive understanding of plant physiology, growth, development, and responses to environmental stress. Traditional plant imaging techniques, such as optical and electron microscopy, are valuable for morphological characterization and chemical localization. However, their reliance on labeling specific chemical compounds limits their applicability to the study of unlabeled and unknown molecules. Mass spectrometry imaging (MSI) offers a significant advantage by enabling label-free, untargeted, and multiplexed analysis of plant metabolites, allowing for de novo chemical discovery and the mapping of their spatial distribution (Feenstra et al. 2017). At present, several MSI methods have been developed for visualizing metabolites within tissues (Kaspar et al. 2011; Boughton et al. 2016), with MALDI being particularly popular due to its broad compound coverage and high spatial resolution (Dong et al. 2016).

Traditional metabolomics approaches, such as LC-MS/MS, require tissue homogenization. This process inevitably leads to the loss of spatial information regarding metabolite localization. In contrast, spatial metabolomics techniques like MALDI-MSI preserve the in situ context of metabolites, enabling both high-resolution localization within specific tissues and dynamic tracking of their temporal changes during biological processes. For example, a spatial metabolomic map of *Ginkgo biloba* leaves revealed that flavonoid ring dimers were specifically enriched in epidermal tissues, whereas ginkgolic acids accumulated in palisade tissues, demonstrating a distinct separation between the biosynthesis and storage sites of secondary metabolites (Li et al. 2018). The spatial metabolomic landscape during strawberry ripening revealed that anthocyanins accumulated in a gradient along the fruit margin, while sugars exhibited global diffusion, elucidating the intrinsic relationship between the spatial distribution of specific metabolites and the formation of fruit color and sweetness (Wang et al. 2021). A spatial metabolomics atlas of tea seedlings revealed a molecular framework for carbon-nitrogen coregulated organogenesis, characterized by a nitrogen flux network where theanine was directionally transported to meristematic tissues via the stele sheath, and spatial regions with pronounced metabolite enrichment derived from cotyledon carbon sources at the root and shoot apices (Fu et al. 2024). Notably, the triterpenoid saponins in tea plants exhibited a “tissue-compartment” dual-dimensional distribution pattern, with cinnamoyl tetraglycoside saponins enriched in aerial tissues and tetraglycoside saponins specifically accumulated in the root stele. This spatial separation offered a valuable model for understanding the complex regulation of secondary metabolite synthesis and transport (Du et al. 2025). In rice seeds, carbohydrates and amino acids were enriched in the seed coat and embryo, while flavonoid precursors diffused into the endosperm. This study provided insights into the metabolic basis of nutritional differences between brown rice and polished rice from a spatial perspective (Zhao et al. 2023). However, current spatial metabolomics research predominantly focuses on plant development, quality, or nutritional metabolism, while the dynamic spatial metabolic alterations during plant–pathogen interactions remain largely unexplored.

Upon pathogen invasion, plants reprogram their metabolic pathways to synthesize diverse defense-related metabolites, including phenolics, alkaloids, and volatile organic compounds. Some of these metabolites exhibit dual functionality, directly suppressing pathogen proliferation while simultaneously inducing systemic resistance in the host plant. For example, small-molecule carbohydrate metabolites like xylose and Myo-inositol, enriched in the rhizosphere of healthy tomato plants, possess dual regulatory capacities. They directly stimulate plant defense signaling and remodel the rhizosphere microbial community by promoting the proliferation of beneficial symbionts that produce antibiotics and compete for resources. Consequently, they effectively restrict ecological niches for pathogens like *Ralstonia solanacearum* and *Verticillium dahliae* (Wen et al. 2023). Similarly, the pepper rhizosphere-associated plant growth-promoting rhizobacteria strain *Pseudomonas sp.* JR48 secretes phenylpyruvate, which activates the host phenylalanine metabolic network. This activation drives phenylpropanoid accumulation and potentiates SA-mediated signaling, ultimately enhancing lignin biosynthesis through peroxidase gene regulation. The resulting lignified physical barrier confers effective resistance against *Phytophthora capsici* infection (Li et al. 2025b). Notably, spatial transcriptomics analysis of arbuscular mycorrhizal interactions has demonstrated significant spatiotemporal heterogeneity in gene expression within plant root cortical cells, revealing a bidirectional gene regulatory network between the host and symbiotic fungi (Serrano et al. 2024). Despite advances in plant–microbe metabolomics, systematic and spatially resolved analyses that link when and where pathogen-responsive metabolites accumulate and diffuse to local resistance and systemic immunity are still lacking, underscoring the need to map their spatiotemporal dynamics during infection.

Here, we combine time-resolved MALDI-MSI spatial metabolomics with physiological characterization and functional validation to dissect the spatiotemporal metabolic basis of tobacco defense against *P. syringae*. Temporally, profiling 1,399 MSI-annotated metabolites across 0, 12, 24, and 60 h postinfection (hpi) reveals a staged metabolic transition, with early responses dominated by organic acids and terpenoids and late infection characterized by pronounced accumulation of alkaloids, accompanied by dynamic remodeling of additional defense-related classes. Spatially, constrained clustering resolves anatomically aligned metabolic compartments and uncovers strong cell type preferences and directional redistribution among epidermal, mesophyll, and vascular regions: epidermal tissues preferentially enrich defense-associated organic acids, alkaloids (notably nicotine), and amino acid-related metabolites with infection-stage dynamics, whereas vascular bundles concentrate hormone-associated signals including SA and jasmonic acid (JA). Finally, exogenous application assays and genetic perturbations of key biosynthetic pathways provide functional support for some key metabolites and JA. SA-associated defense contributes to limiting lesion development and bacterial proliferation. Together, this work establishes a spatiotemporal metabolic atlas of tobacco wildfire disease progression and offers a framework for prioritizing defense metabolites and pathways for mechanistic dissection and resistance improvement.

## Materials and methods

### Plant materials

The seeds of *Nicotiana tabacum* (cv. K326) were surface-sterilized and grown on half-strength Murashige and Skoog (1/2 MS) solid medium supplemented with 3% sucrose. 1 wk after germination, the seedlings were transplanted into individual pots and grown under a 28 °C/25 °C day/night cycle with a 16-h light/8-h dark photoperiod. At 21 d post-transplantation during the 6-leaf stage, they were inoculated with *P. syringae*, which was collected from the Guizhou Academy of Tobacco Science. The *P. syringae* strain was cultured in antibiotic-free LB liquid medium at 28 °C and then gently resuspended in phosphate-buffered saline (PBS) buffer to an optical density value of 0.001. The prepared bacterial suspension was injected into the abaxial side of tobacco leaves using a disposable sterile syringe. Leaf samples from infected tobacco plants were collected at 4 time points: 0, 12, 24, and 60 hpi, with 3 biological replicates per time point. Representative disease phenotype images were selected from 1 of 3 biological replicates. Due to the parallel execution of these assays under identical inoculation and imaging conditions, the representative images from the same wild-type cytokinin (CK) group were employed as a shared control across all presented panels (Figs. 5c, 6c, and Figure S4b). The samples were placed in embedding molds (with sample orientation labeled on the lid) and fully embedded in a 2% (m/v) carboxymethyl cellulose solution. The embedded samples were then frozen in a dry ice-ethanol mixture. Once the embedding medium turned completely white, the molds were removed and stored at −80 °C for spatial metabolomics analysis.

### MALDI sample preparation

During sectioning, the tissues were stabilized with 3 drops of distilled water and sectioned at −20 °C using a Leica CM1950 cryostat (Leica Microsystems GmbH, Wetzlar, Germany) at a thickness of 50 *μ*m. This thickness was selected as an optimized compromise between maintaining tissue integrity, providing sufficient material for MALDI signal acquisition, and preserving the spatial resolution required for visualization of leaf tissue structures. The tissue sections were then mounted onto indium tin oxide-coated conductive slides and dried in a vacuum desiccator for 30 min.

For matrix application, 2,5-dihydroxybenzoic acid (DHB) was prepared at 15 mg/mL in acetonitrile:water (90:10, v/v). DHB was selected because of its broad applicability and well-established performance in plant spatial metabolomics. In positive ion mode, it efficiently ionizes amino acids and choline-type molecules, whereas in negative ion mode it supports sensitive detection of fatty acids and nucleotides. Its favorable crystallization properties and high efficiency for small-molecule detection make DHB 1 of the most widely used matrices for plant metabolite imaging. The matrix was sprayed at 60 °C, at a flow rate of 0.1 mL/min, and a pressure of 6 psi. Each slide was sprayed 25 times with a 5 s drying interval between sprays (Zeng et al. 2021).

### Image detection and mass spectrometry analysis

MSI was performed by Metware Biotechnology Co., Ltd. (Wuhan, China) using a Bruker timsTOF fleX system (Bruker Daltonics, Bremen, Germany) equipped with a 10 kHz smartbeam 3D laser. MSI data were acquired using flexImaging software (Bruker Daltonics). The laser energy was set to 80%, and each pixel spectrum was accumulated over 400 laser shots. Data were acquired in positive ion mode over an *m/z* range of 50 to 1,300, with a mass resolution of 40,000. The spatial resolution for tissue imaging was set to 50 *μ*m. Before each MALDI-MSI experiment, the mass axis of the instrument was calibrated using a standard tune mix according to the manufacturer's instructions.

Raw MSI data were imported into SCiLS Lab software (Bruker Daltonics) for visualization and root mean square normalization. The normalized image signal intensity was then used to represent the relative abundance of metabolites in the corresponding whole tissue region. For cell-type-specific analysis, the normalized image signal intensity of each annotated tissue region (vascular, mesophyll, and epidermis) represented the relative abundance of metabolites among different cell types. The corresponding relative abundance values are provided in Table S1.

### Metabolite annotation

Metabolite annotation of the MALDI-MSI dataset was performed using a 2-step strategy similar to previous studies (Zhang et al. 2025b; Li et al. 2025c). Preliminary annotation of all metabolite-associated features was achieved by accurate mass matching against a comprehensive reference resource. This resource comprised public metabolite databases (HMDB, KEGG, and METLIN) and internal fragment libraries derived from authentic standards analyzed on both the timsTOF fleX platform and by LC–MS. Mass matching was conducted with a tolerance of ±10 ppm (Fu et al. 2024; Mao et al. 2024; Zhang et al. 2025a).

To enhance annotation confidence, metabolite-associated features with relatively high signal intensity were further subjected to on-tissue tandem mass spectrometry (MALDI-MS/MS) analysis after MSI acquisition. Ions of interest were isolated using a ±2 Da window and fragmented by collision-induced dissociation with collision energies ranging from 10 to 40 eV. The acquired spectra were recalibrated using the single-point calibration option and reprocessed using DataAnalysis software (version 5.3; Bruker Daltonics, Bremen, Germany).

Experimental MS/MS spectra were compared with the comprehensive reference resource described above. First, MS/MS data were extracted and aligned with the MS1 peak table to associate valid MS/MS spectra with their corresponding precursor ions. For each valid MS/MS spectrum, candidate metabolites were retrieved from the reference resource based on precursor ion information. Subsequently, spectral matching was performed by jointly considering precursor ion agreement (Q1), retention time (when available), and MS/MS spectral similarity. The matching score was calculated using a weighted scoring strategy, with MS/MS similarity as the major component, following the principle of spectral entropy-based similarity scoring for small-molecule library searching (Li et al. 2021). Candidate annotations exceeding the default confidence threshold were retained, and redundant hits were removed to generate the final nonredundant annotation list. Metabolite-associated features supported by on-tissue MS/MS spectral matching were assigned as Level 2 annotations according to the Metabolomics Standards Initiative guidelines (Sumner et al. 2007), whereas features lacking informative MS/MS spectra were classified as putative annotations based on accurate mass matching, with their “MS level” indicated in Table S1.

### Targeted LC–MS/MS validation of phytohormones

HPLC-grade acetonitrile and methanol (Merck) and Milli-Q water were used throughout. Authentic standards were purchased from OlChemIm Ltd. and isoReag, and stock solutions (1 mg/mL in methanol) were stored at −20 °C prior to use. For extraction, fresh tobacco tissues were harvested, immediately frozen in liquid nitrogen, ground to a fine powder, and stored at −80 °C. Approximately 50 mg of tissue powder was extracted with 1 mL methanol/water/formic acid (15:4:1, v/v/v) containing 10 *μ*L of internal standard mixture (100 ng/mL). After vortexing for 10 min and centrifugation at 12,000 rpm for 5 min at 4 °C, the supernatant was collected, evaporated to dryness, reconstituted in 100 *μ*L of 80% methanol, and filtered through a 0.22 *μ*m membrane prior to LC–MS/MS analysis.

Targeted LC–MS/MS analysis was also performed by Metware Biotechnology Co., Ltd. (Wuhan, China) using an ExionLC AD UPLC system coupled to a QTRAP 6500+ mass spectrometer (SCIEX). Chromatographic separation was achieved on a Waters ACQUITY UPLC HSS T3 C18 column (100 mm × 2.1 mm, 1.8 *μ*m). The mobile phases consisted of water containing 0.04% acetic acid (A) and acetonitrile containing 0.04% acetic acid (B), with the following gradient program: 5% B (0 to 1 min), 5% to 95% B (1 to 8 min), 95% B (8 to 9 min), and re-equilibration to 5% B (9.1 to 12 min). The flow rate was 0.35 mL/min, the column temperature was maintained at 40 °C, and the injection volume was 2 *μ*L.

The QTRAP 6500+ instrument was operated in both positive and negative electrospray ionization modes, with a source temperature of 550 °C, ion spray voltage of 5,500 V/−4,500 V, and curtain gas of 35 psi. Quantification of phytohormones was performed using scheduled multiple reaction monitoring (MRM), and compound-specific declustering potentials and collision energies were optimized for individual MRM transitions. Data acquisition and processing were conducted using Analyst 1.6.3 and MultiQuant 3.0.3 (SCIEX).

### *P. syringae* DNA extraction and quantification in inoculated leaves

To determine the biomass of *P. syringae* in tobacco leaves after infection, samples were collected from infected spots at each time point. Each sample weighed approximately 100 mg, with 3 biological replicates per treatment. Genomic DNA was extracted using a plant genomic DNA extraction kit (TIANGEN, Beijing, China). The pathogen content was quantified by qPCR using *P. syringae*-specific primers targeting the tabtoxin biosynthetic gene (tabB) (tabB1F: TGATTCTGCTGTCCTTTCTAAC; tabB1R: ATGCAAGCGTCACGAAACGC) (Debode et al. 2009).

### Mfuzz clustering and differentially expressed metabolite screening

Mfuzz soft clustering was performed in R using the Bioconductor package Mfuzz, which applies fuzzy c-means clustering to dynamic omics datasets (Kumar and Futschik 2007). The regions of interest (ROI)-resolved metabolite abundance matrix was used as the input. For tissue distribution pattern analysis, metabolite abundance profiles across the epidermis, mesophyll, and vascular region were clustered into 6 groups. For time-course pattern analysis, selected metabolite abundance profiles across 0, 12, 24, and 60 hpi were clustered into 4 groups.

Fold-change-based metabolite screening was performed using the normalized MALDI-MSI intensity matrix. For each metabolite, fold changes were calculated by comparing its abundance at 12, 24, and 60 hpi against that at 0 hpi. In the absence of replicate-level measurements, metabolites with log_2_FC ≥ 1 were defined as differentially expressed metabolites (DEMs).

## Results

### *P. syringae* infection significantly affects tobacco growth and cellular structure

**Figure 1:**
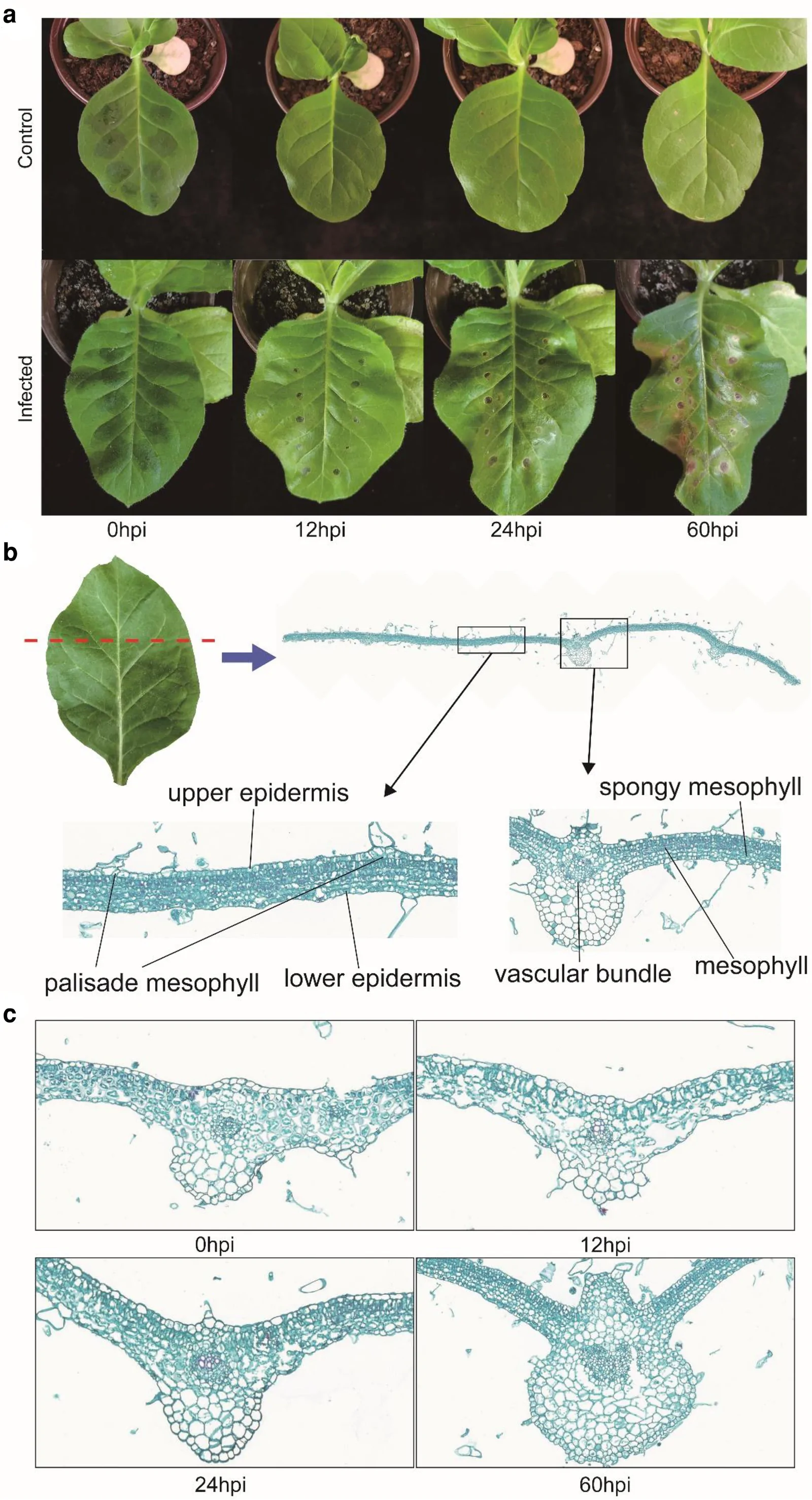
Phenotypic and microscopic structural changes in tobacco leaves at different infection time points of *Pseudomonas syringae*. a) Representative phenotypes of tobacco leaves from control (PBS) and *P. syringae*-infected plants at different time points postinoculation. b) Cross-sectional anatomical structure of tobacco leaves. c) Microscopic structural changes in cross-sections of tobacco leaves at different infection time points.

To investigate the effects of *P. syringae* infection on tobacco growth, 30-d-old tobacco seedlings were subjected to pathogen infection. At 12 hpi, no significant symptoms were observed at the infection sites, and in contrast to PBS-infiltrated controls that remained symptom-free, localized light brown lesions appeared at 24 hpi and developed into characteristic dark brown necrotic areas at 60 hpi (Fig. 1a and Figure S1a). The 60 hpi infected leaf image in Figure S1a corresponds to the same representative infection phenotype shown in Fig. 1a and was reused to indicate the sampling regions for bacterial quantification; this analysis showed that bacterial abundance was highest in the lesion center, lower in boundary tissues, and lowest in distal tissues, demonstrating a spatial gradient of pathogen accumulation at the severe infection stage. As stomata serve as major entry points for pathogens into host tissues, their dynamic opening and closure play a crucial role in plant defense against pathogen invasion. Therefore, we examined the structural features and aperture status of stomata during infection (Figure S1b and c). Scanning electron microscopy revealed that stomatal morphology and aperture underwent marked changes as infection progressed at 12 and 24 hpi, stomatal apertures significantly increased, whereas by 60 hpi, stomatal closure became evident. Quantitative analysis further demonstrated that stomatal length, width, and aperture percentage varied across different time points, with significantly higher aperture percentages observed at 12 hpi and 24 hpi compared with those at 0 and 60 hpi. These results indicated that dynamic stomatal regulation was closely associated with pathogen entry and spread.

To examine cellular structural changes in tobacco following pathogen infection, paraffin-embedded sections of infected samples were prepared at different time points and analyzed using an optical microscopy system (Fig. 1b). The results revealed dynamic cytological responses in tobacco mesophyll cells. Initially (0 hpi), the palisade and spongy cells exhibited a tightly arranged polar structure. Between 12 and 24 hpi, intercellular spaces significantly increased, and cell arrangement displayed irregular polarity. By 60 hpi, the intercellular spaces had returned to their initial state, and the overall cell arrangement closely resembled that observed at 0 hpi, with progressive changes noted between 12, 24, and 60 hpi. This restoration of cellular structure might be associated with the spatiotemporal activation of plant cell wall remodeling or defense responses (Fig. 1c).

### Temporal dynamics of metabolic reprogramming during *P. syringae* infection

To gain a more precise understanding of the impact of pathogen infection on plant metabolites, MALDI-MSI-based spatial metabolomics was employed to systematically analyze metabolites in tobacco leaves at 0, 12, 24, and 60 hpi following *P. syringae* infection.

**Figure 2:**
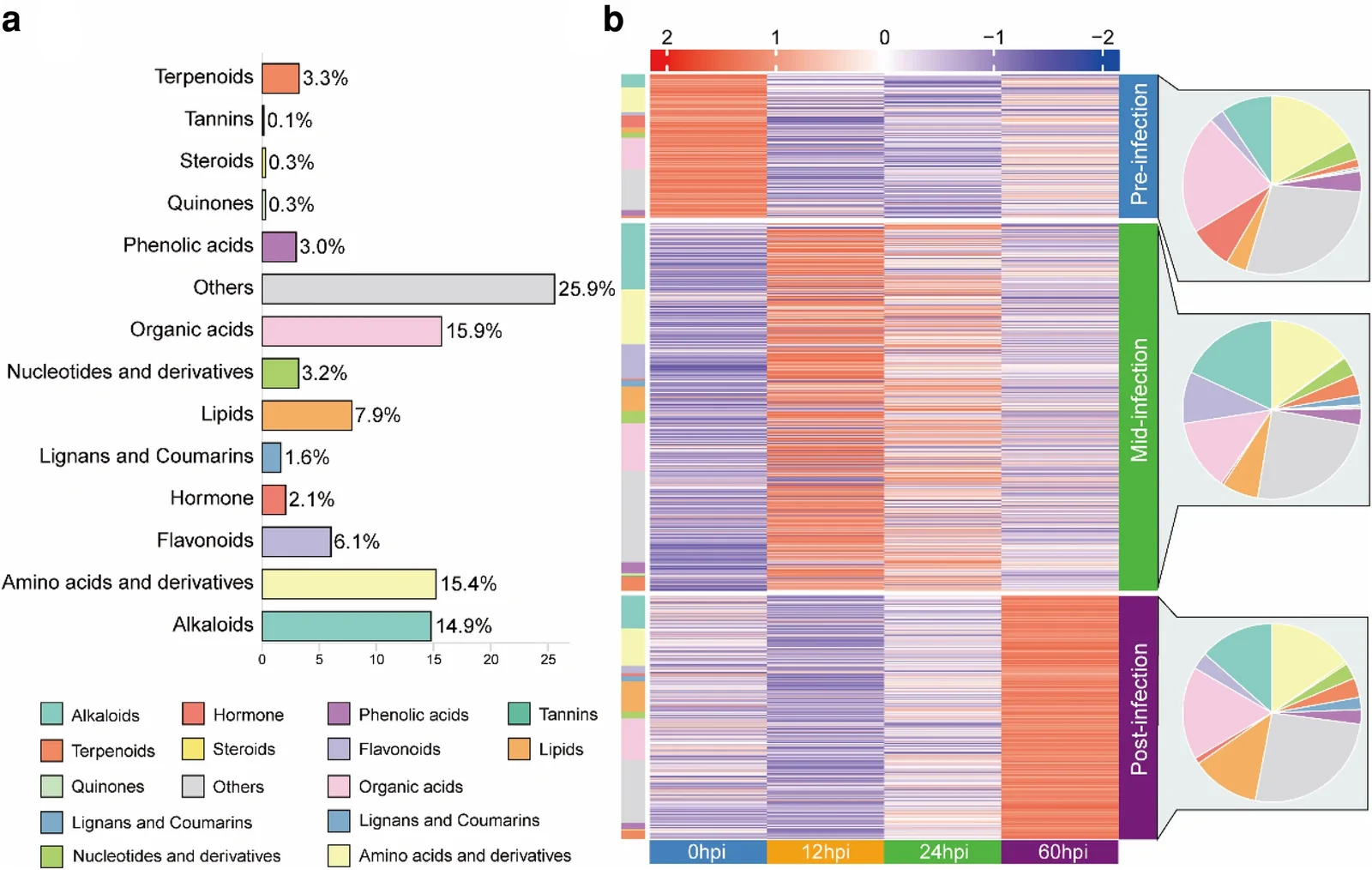
Global metabolomic profiling of tobacco leaves in response to *Pseudomonas syringae* infection. a) Classification of all detected metabolites in the samples. b) Heatmap of the relative abundance of all metabolites.

A total of 1,399 metabolite-associated MSI features were detected and putatively annotated. These metabolites were subsequently classified into 14 major categories: amino acids and their derivatives, phenolic acids, nucleotides and their derivatives, hormones, flavonoids, quinones, lignans and coumarins, tannins, alkaloids, terpenes, organic acids, lipids, steroids, and other compounds (Fig. 2a and Table S1). Among them, organic acids, amino acids and derivatives, and alkaloids were present in relatively high amounts, accounting for 15.9%, 15.4%, and 14.9%, respectively.

By comparing the spatial metabolite profiles across different infection time points using relative abundance on whole tissue level, we found that numerous metabolites exhibited pronounced temporal reprogramming during pathogen invasion, and this response became progressively stronger as infection advanced (Fig. 2b). Although hormone-related metabolites accounted for a relatively small proportion of the total metabolite pool, they displayed a distinct early-stage response, indicating that phytohormone-mediated signaling was rapidly activated during the initial phase of pathogen challenge and likely played a critical regulatory role in tobacco defense. In contrast, most amino acids and their derivatives, lipids, and alkaloid-related metabolites accumulated predominantly at 60 hpi, suggesting that these metabolite classes are closely associated with the later defense phase. This pattern further implies that amino acids and their derivatives may serve as important metabolic precursors for the biosynthesis of antimicrobial compounds, including alkaloids, thereby enhancing the defensive capacity of infected tissues. In addition, flavonoid- and terpenoid-related metabolites exhibited bidirectional regulatory patterns during infection, reflecting a dynamic redistribution of defense-associated metabolic resources across different stages of pathogen induction (Fig. 2b).

### Spatial metabolic heterogeneity and compartmentalized defense responses during *P. syringae* infection

**Figure 3:**
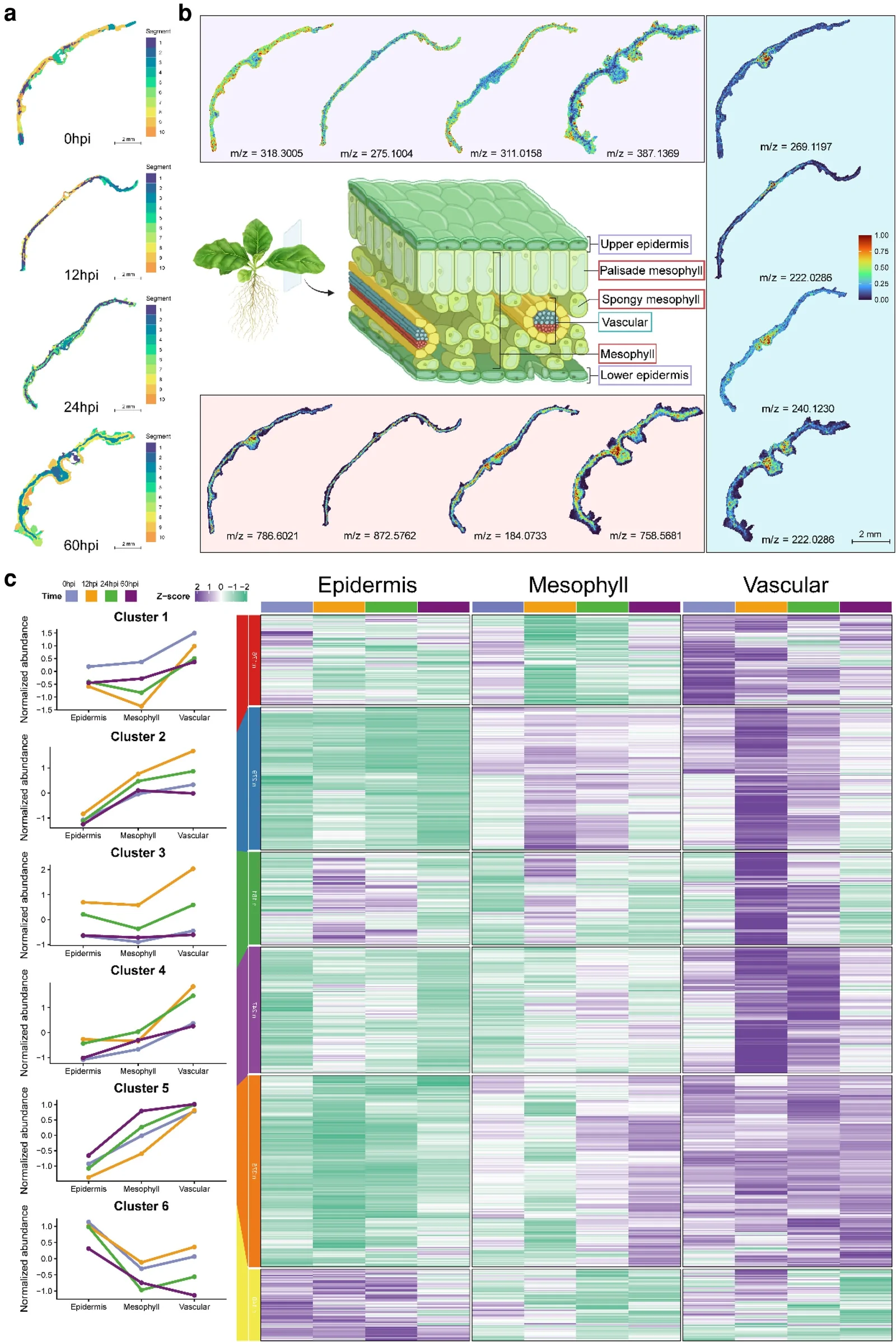
MALDI-MSI reveals spatial heterogeneity and coordinated spatiotemporal metabolic reprogramming during *Pseudomonas syringae* infection. a) Spatial clustering-based segmentation maps of leaf sections at 0, 12, 24, and 60 hpi derived from global metabolite intensity patterns. b) Representative ion images showing cell type–preferential spatial distribution patterns across infection stages. Note: The specific ionization modes and adduct forms employed for compound identification are explicitly indicated in the main text. c) Heatmap showing metabolite abundance patterns across epidermis, mesophyll, and vascular regions at 0, 12, 24, and 60 h, with line plots indicating the average tissue-wise abundance patterns at each time point.

MALDI-MSI enabled direct visualization of infection-induced metabolic heterogeneity in tobacco leaves across 0, 12, 24, and 60 hpi. To quantitatively characterize metabolite distribution across major anatomical regions, ROIs were manually annotated on the MSI images according to leaf anatomical features and corresponding tissue morphology. 3 major tissue regions, including the epidermis, mesophyll, and vascular region, were defined as ROIs for subsequent cell type-resolved spatial quantification. For each ROI, the relative abundance of each metabolite was represented by the normalized intensity of the corresponding target ion, with higher signal intensity indicating higher relative metabolite abundance in that region. This ROI-based spatial analysis revealed clear compartmentalized metabolic organization across the major leaf tissue regions and showed that subtissue-level metabolic patterns were dynamically remodeled as infection progressed (Fig. 3a). Representative ion images further illustrated distinct tissue-preferential distribution patterns, with specific ions predominantly localized to the epidermal, vascular, or mesophyll regions, and these spatial distributions changed across infection stages, highlighting pronounced spatiotemporal heterogeneity in the metabolic response (Fig. 3b).

To further characterize these spatially patterned dynamics at a cell type-resolved scale, we compared the MALDI-MSI-derived relative abundances of metabolites across the epidermis, mesophyll, and vascular region at 0, 12, 24, and 60 hpi. The cell type-resolved metabolite abundance matrix was subjected to soft clustering analysis using the Mfuzz package, which implements fuzzy c-means clustering for omics time-series and dynamic pattern analysis. This analysis revealed pronounced tissue-associated metabolic patterns together with strong infection-stage-dependent remodeling after *P. syringae* infection, and 6 major spatial clusters with distinct tissue distribution patterns were identified for all detected metabolites (Fig. 3c). Among them, spatial Cluster 5 exhibited a progressive increase in relative abundance from the epidermal region toward the vascular region as infection advanced, indicative of an outside-to-inside redistribution of metabolic responses. Conversely, spatial Cluster 6 displayed the opposite trend, suggesting an inside-to-outside redistribution during infection (Fig. 3c). Because these 2 clusters represented the most prominent directional redistribution patterns across leaf tissue regions, metabolites assigned to spatial Clusters 5 and 6 were further extracted and subjected to a separate time-course Mfuzz clustering analysis to characterize their temporal response patterns during infection. This temporal clustering analysis classified these metabolites into 4 time-dependent response patterns. Among them, temporal Cluster 3 was characterized by an early-induced but late-repressed pattern, whereas temporal Cluster 4 showed a continuously accumulating pattern across the infection time course (Figure S2a).

Based on the metabolites resolved by the spatial and temporal Mfuzz clustering analyses, we further performed fold-change-based differential screening to identify metabolites with infection-stage-dependent abundance changes. This screening revealed pronounced stage specificity, with a core set of 28 DEMs consistently detected at 12, 24, and 60 hpi relative to 0 hpi (Figure S2b). These shared DEMs were mainly composed of organic acids and alkaloids, followed by amino acids and their derivatives (Figure S2b), and showed coordinated temporal dynamics together with cell type-preferential spatial distributions (Figure S2c).

Collectively, these results indicate that *P. syringae* infection triggers a coordinated spatiotemporal reprogramming of tobacco metabolism, characterized by ROI-defined anatomical compartmentalization, directional cell-type-scale redistribution, and stage-specific accumulation changes of infection-responsive metabolites throughout the infection process.

### Unraveling spatiotemporal dynamics of key defense metabolites during *P. syringae* infection

To identify infection-responsive metabolites and characterize their spatiotemporal dynamics during *P. syringae* infection, we performed fold-change-based differential screening using the relative abundance matrix derived from MALDI-MSI, with 12, 24, and 60 hpi samples each compared with 0 hpi as the reference. We then examined the screened DEMs together with their spatial distribution patterns to reveal how metabolic responses were dynamically remodeled across infection stages and tissue regions. This analysis revealed clear stage-dependent changes in metabolite accumulation throughout the infection timeline.

**Figure 4:**
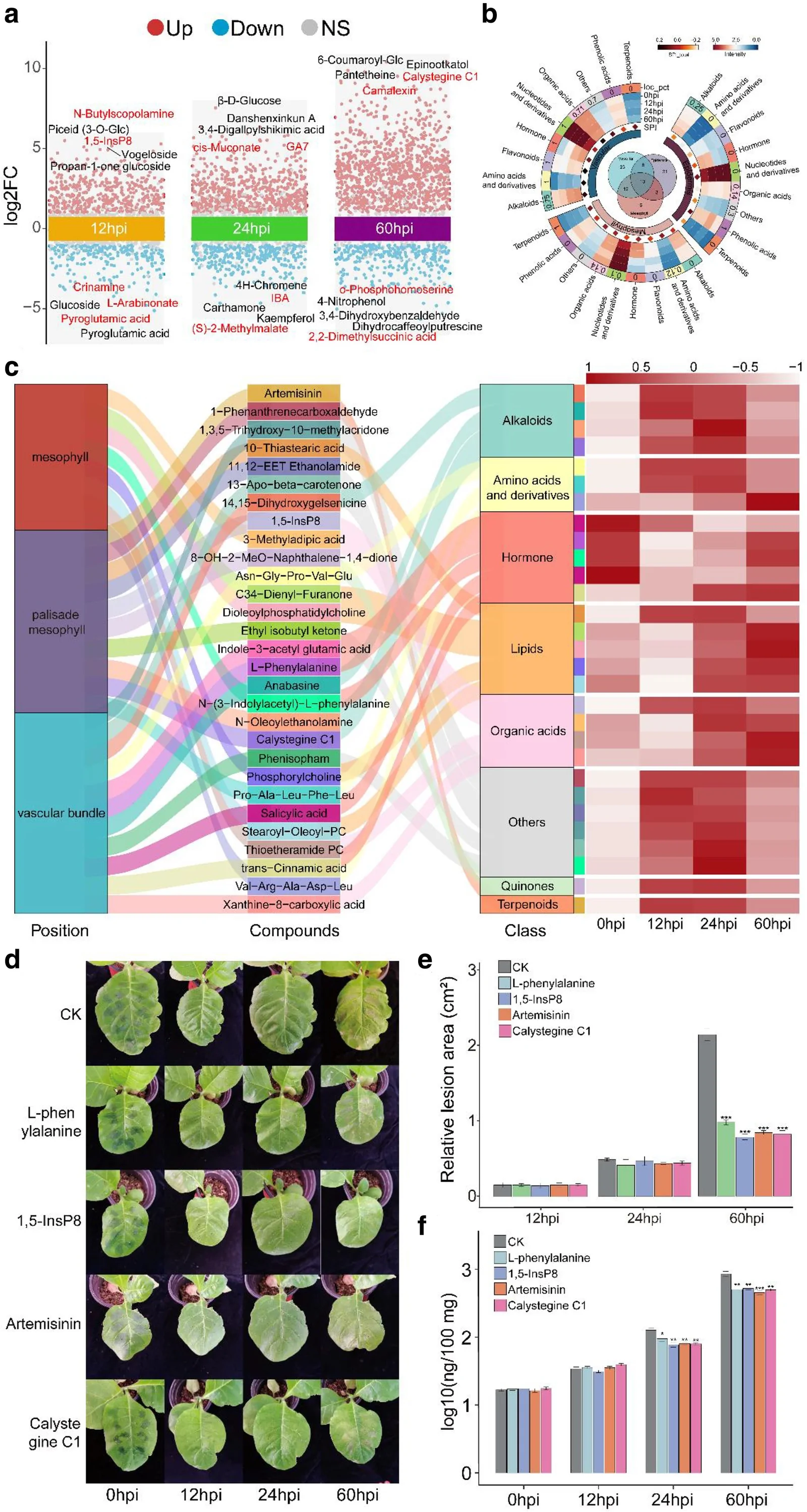
Key defense metabolites in tobacco leaves during *Pseudomonas syringae* infection. a) Manhattan plots showing differentially expressed metabolites (DEMs) at 12 hpi, 24 hpi, and 60 hpi relative to 0 hpi. Metabolites classified as hormones, alkaloids, organic acids, and amino acids and derivatives are specifically indicated for clarity. b) Circular heatmap summarizing features of DEMs, including Intensity (mean abundance), loc_pct (proportion of DEMs in each cell type), and SPI_total (overall regulation trend relative to 0 hpi across postinfection time points), together with a Venn diagram showing the overlap of DEMs among epidermis, mesophyll, and vascular regions. c) The spatial location information for key DEMs. d) Representative phenotype of tobacco leaves pretreated with exogenous metabolites (L-phenylalanine, 1,5-InsP8, Artemisinin, or Calystegine C1) during *P. syringae* infection, with CK as the mock-treated control. e) Quantification of relative lesion area in CK and treated plants at the indicated time points. f) Quantification of *P. syringae* DNA levels in CK and treated plants at the indicated time points (log10 ng pathogen DNA per 100 mg leaf tissue). Data are means ± SEM from 3 independent biological replicates (*N* = 3). Statistical significance in panels B and E was evaluated using 2-tailed Student's *t*-tests (*P* < 0.05 (*), *P*  *<*  *0.01* (**), *P*  *<*  *0.001* (***); NS, not significant). For multiple-group comparisons in panel D, 1-way ANOVA followed by the Tukey–Kramer test was used; different lowercase letters above bars indicate significant differences at *P* < 0.05.

At the early infection stage (12 hpi), organic acids and terpenoids, including 1,5-InsP8 and vogeloside, showed increased abundance, whereas lipids, amino acids, and their derivatives, including methyl 12-phenyldodecanoate and pyroglutamic acid, were predominantly decreased. By 24 hpi, the dominant increased metabolite classes shifted toward phenolic acids and quinones, such as 3,4-digalloylshikimic acid and danshenxinkun A, while flavonoids, such as carthamon, showed decreased abundance. By 60 hpi, alkaloids became the predominant class of metabolites with increased abundance, with Calystegine C1 and camalexin among the representative metabolites, whereas several organic acids, including 2,2-dimethylsuccinic acid and 3,4-dihydroxybenzaldehyde, showed decreased abundance (Fig. 4a). Temporal trends indicated that most log_2_FC-defined increased metabolites reached their highest abundance at 60 hpi, whereas decreased metabolites were more frequently observed at 24 and 60 hpi (Figure S3a). Moreover, the number of log_2_FC-defined increased metabolites was higher than that of decreased metabolites, with alkaloids, amino acids and their derivatives, and phenolic acids being highly represented among the increased metabolites, and flavonoids and terpenoids being more frequently represented among the decreased metabolites (Figure S3b).

To contextualize these temporal dynamics within the anatomical structure of the leaf, we constructed a circular heatmap to summarize metabolite changes at the metabolite-class level. For each metabolite class, the visualization integrated 3 metrics: mean normalized intensity in each tissue region, loc_pct (the proportion of differentially accumulated metabolites, DEMs, within that class contributed by each cell type), and SPI_total (an integrated index summarizing the overall regulation trend of that metabolite class across postinfection time points relative to 0 hpi) (Fig. 4b). This integrative view revealed pronounced cell type-biased regulation at the metabolite-class level, emphasizing the compartmentalized nature of the plant's metabolic response rather than a uniform, leaf-wide reprogramming. Overlap analysis of DEMs among epidermis, mesophyll, and vascular regions confirmed substantial cell type specificity together with a smaller shared core set, supporting a coordinated yet spatially distinct defense program (Fig. 4b). Building on the metabolite class-level patterns and their cell type distributions, we next identified representative metabolites showing preferential accumulation in specific cell types in order to define the key chemical features associated with spatial heterogeneity. Spatial mapping showed that major metabolite classes displayed distinct and dynamic enrichment patterns in epidermal, mesophyll, and vascular regions across infection stages. Based on these patterns, we further identified representative metabolites that characterized epidermis-, mesophyll-, or vascular-associated defense programs (Fig. 4c).

To experimentally validate the functional roles of these key metabolites in enhancing tobacco resistance, we performed exogenous pretreatment experiments. Treatment with L-phenylalanine, 1,5-InsP8, artemisinin, and calystegine C1 each significantly enhanced tobacco resistance to *P. syringae*. Compared with the control (CK), treated plants displayed attenuated lesion symptoms (Fig. 4d) and a smaller lesion area at 60 hpi (Fig. 4e). Pathogen biomass quantification confirmed that these treatments effectively suppressed bacterial proliferation at 24 or 60 hpi, resulting in reduced *P. syringae* DNA levels (Fig. 4f). These functional validations were also supported by existing literature. L-phenylalanine was a key precursor of defense phenolics (Dixon et al. 2002); 1,5-InsP8 (Laha et al. 2015), an inositol pyrophosphate, was linked to JA-associated immunity; artemisinin exhibited antimicrobial activity (Appalasamy et al. 2014); and calystegine C1 was a known defense alkaloid (Goldmann et al. 1996). In conclusion, these analyses indicate that tobacco metabolism is reprogrammed in a strongly stage-dependent and spatially compartmentalized manner during *P. syringae* infection. Consistently, exogenous application of selected candidates reduced lesion development and bacterial proliferation, supporting their functional contribution to tobacco resistance.

### Hormones mainly dynamically respond to *P. syringae* infection in vascular bundles

**Figure 5:**
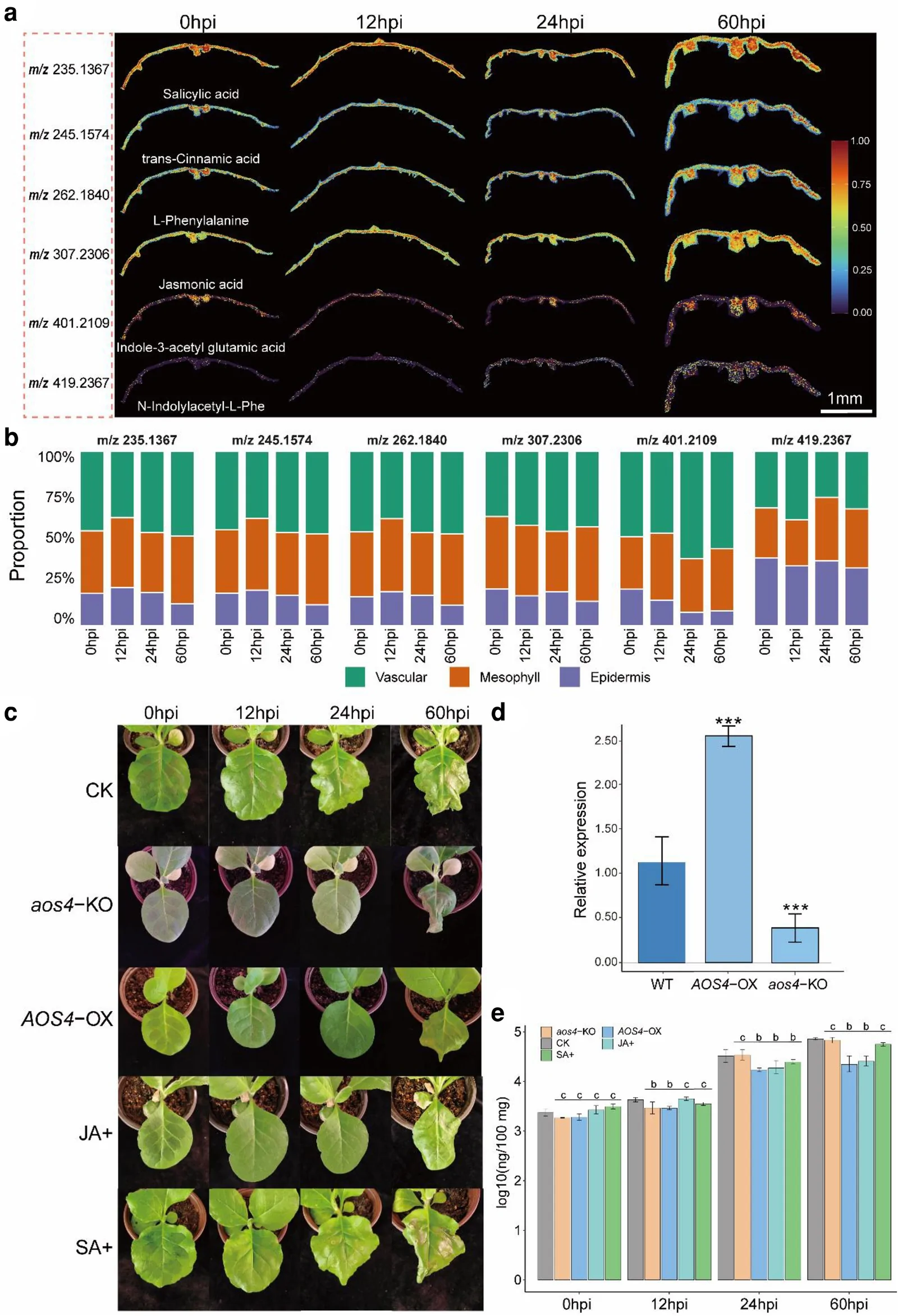
Spatiotemporal distribution of hormone metabolites and functional validation of SA/JA-mediated defense responses during *Pseudomonas syringae* infection in tobacco. a) Positive-mode MALDI images of each selected hormone ion. Each column presents ion images of corresponding compounds at different infection stages. The intensity scale represents relative abundance, with scale variation indicating differences in relative ion intensity within the region. Scale bar = 1 mm. b) The proportions of various typical metabolites in different leaf cell types, including mesophyll cells, epidermal cells, and vascular cells. c) Phenotype of wild-type control (CK), *aos4*-KO, *AOS4*-OX, JA-treated (JA+), and SA-treated (SA+) plants at 0, 12, 24, and 60 hpi. The same representative wild-type CK images were used as the shared CK control in Fig. 5c, Fig. 6c, and Figure S4B. d) Relative expression of *AOS4* in WT, *AOS4*-OX and *aos4*-KO lines. e) Quantification of bacterial populations in leaves (log_10_ ng pathogen DNA per 100 mg) at 0, 12, 24, and 60 hpi. Data are means ± SEM from 3 independent biological replicates (*N* = 3). Statistical significance in panel D was evaluated using 2-tailed Student's *t*-tests (*P* < 0.05 (*), *P*  *<*  *0.01* (**), *P*  *<*  *0.001* (***); NS, not significant). For multiple-group comparisons in panel E, 1-way ANOVA followed by the Tukey–Kramer test was used; different lowercase letters above bars indicate significant differences at *P* < 0.05.

Plant hormones are endogenous small molecules that play a central role in regulating growth, development, and responses to environmental stress, and trigger the plant immune signaling network upon pathogen invasion (Katagiri and Tsuda 2010). While SA and JA are well-established defense hormones, ethylene, abscisic acid (ABA), gibberellins, auxins, and cytokinins (CKs) also contribute to plant immunity against diverse pathogens (Robert-Seilaniantz et al. 2011; Shigenaga et al. 2017). To further investigate the dynamic spatial and temporal changes of plant hormones during pathogen infection, we employed MALDI-MSI-based spatial metabolomics to characterize hormone distribution in tobacco at 4 infection time points. The results revealed that most hormones were primarily enriched in the vascular bundles, with a smaller portion accumulating in the spongy mesophyll cells, and exhibited significant content variations across different infection stages (Fig. 5a). Specifically, SA and JA levels showed a slight decrease during the early stages of pathogen infection, followed by a gradual increase in later stages, indicating that tobacco might initially prioritize rapid activation of defense mechanisms to minimize signal interference or evade pathogen effector proteins (Zhao et al. 2024). As the infection progresses, the plant gradually activates hormone-mediated systemic resistance through recognition of pathogen-specific signals, establishing a multilayered defense system (Li et al. 2025a). Auxin distribution was also concentrated in the vascular bundles and spongy mesophyll cells, exhibiting distinct temporal characteristics. Among them, precursor metabolites related to auxin synthesis (such as trans-Cinnamic acid, L-Phenylalanine, and Indole-3-acetyl glutamic acid) showed a transient decrease in abundance during the early stages of infection, followed by a subsequent increase, while the auxin derivative N-Indolylacetyl-L-Phe exhibited a continuous increase after pathogen infection. Notably, unlike most hormone-related metabolites that were mainly concentrated in vascular bundles and mesophyll, N-Indolylacetyl-L-Phe showed a marked enrichment in epidermal cells (Fig. 5b and Figure S4a). This dynamic behavior likely reflects the complex regulatory mechanisms governing the auxin metabolic pathway during pathogen infection, potentially linking the balance between plant growth and immunity with the integration of defense signals.

To functionally validate the SA/JA-associated defense roles inferred from the hormone dynamics, we performed exogenous hormone treatments and genetic manipulations of key genes in the JA biosynthetic pathway. Compared with CK, exogenous JA pretreatment consistently alleviated disease symptoms across infection stages and significantly reduced bacterial accumulation, and SA pretreatment also lowered pathogen biomass (Fig. 5c and e). In parallel, we investigated the role of *AOS4*, a core enzyme in JA biosynthesis. Overexpression of *AOS4* (*AOS4*-OX) led to increased gene expression (Fig. 5d) and attenuated lesion development (Fig. 5c), whereas *AOS4* knockout (*aos4*-KO) lines exhibited reduced expression (Fig. 5d) and more severe disease symptoms (Fig. 5c). Pathogen quantification further corroborated these observations, showing lower *P. syringae* DNA levels in *AOS4*-OX plants and higher bacterial loads in *aos4*-KO plants compared with CK (Fig. 5e). Similarly, *LOX* overexpression lines, targeting another key step of JA biosynthesis, also displayed enhanced resistance, manifested by reduced symptom severity, smaller lesion areas, and decreased pathogen biomass compared with CK (Figure S4b, c and d). Collectively, the exogenous and genetic validation results highlight the predominant protective role of JA/SA biosynthesis in restricting *P. syringae* proliferation.

### Alkaloids, organic acids, and amino acids and their derivatives enriched in epidermal cells exhibit dynamic responses to *P. syringae* infection

**Figure 6:**
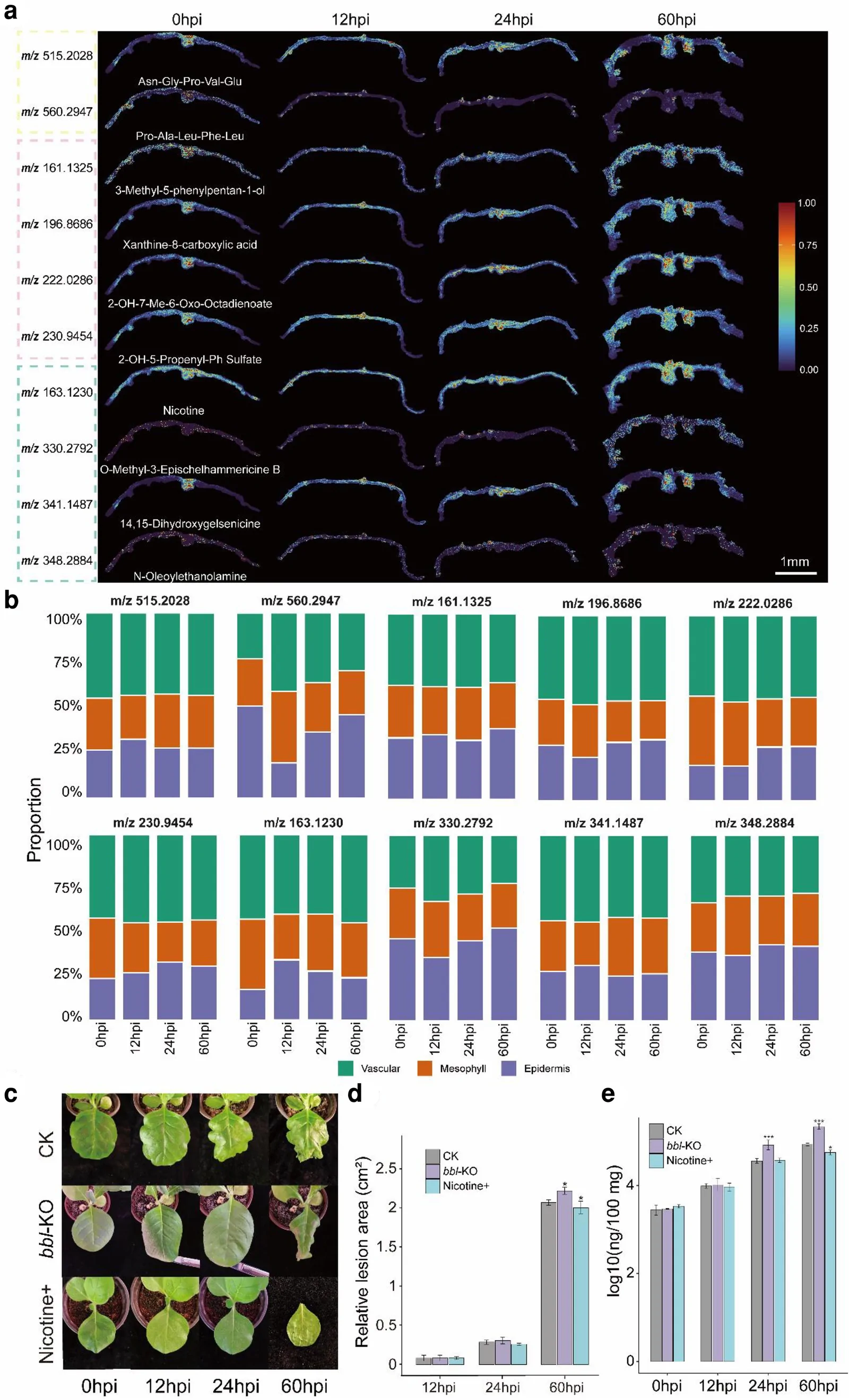
Spatially resolved distribution of defense-associated metabolites and functional validation of nicotine-mediated resistance to *Pseudomonas syringae* in tobacco. a) Positive-mode MALDI images of each selected alkaloid, organic acid, amino acid, and their derivative ions. Each column presents ion images of corresponding compounds at different infection stages. The intensity scale represents relative abundance, with scale variation indicating differences in relative ion intensity within the region. Scale bar = 1 mm. b) The proportions of various typical metabolites in different leaf cell types, including mesophyll cells, epidermal cells, and vascular cells. c) Representative disease phenotype of wild-type control (CK), the low-nicotine mutant *bbl* knockout line (*bbl*-KO), and wild-type plants pretreated with exogenous nicotine (Nicotine+) at 0, 12, 24, and 60 hpi. The same representative wild-type CK images were used as the shared CK control in Fig. 5c, Fig. 6c, and Figure S4B. d) Quantification of relative lesion area in CK, *bbl*-KO, and Nicotine+ plants at the indicated time points. e) Quantification of *P. syringae* biomass in leaves of CK, *bbl*-KO, and Nicotine+ plants at 0, 12, 24, and 60 hpi (log10 ng pathogen DNA per 100 mg leaf tissue). Data are presented as log10 (ng pathogen DNA/100 mg leaf tissue). Data are means ± SEM from 3 independent biological replicates (*N* = 3). Statistical significance in panels D and E was evaluated using 2-tailed Student's *t*-tests (*P* < 0.05 (*), P  *<*  0.01 (**), P  *<*  0.001 (***); NS, not significant).

MALDI-MSI-based spatial metabolomics analysis revealed that specific organic acids (eg 3-Methyl-5-phenylpentan-1-ol, Xanthine-8-carboxylic acid, 2-OH-7-Me-6-Oxo-Octadienoate, and 2-OH-5-Propenyl-Ph Sulfate) were enriched in the epidermal cells of tobacco leaves. The levels of these organic acids exhibited a dynamic pattern of initial decrease, followed by an increase during pathogen infection, suggesting rapid consumption during early pathogen invasion, followed by compensatory synthesis regulated by plant metabolism in later stages. Furthermore, following pathogen infection, most alkaloids (eg 14,15-Dihydroxygelsenicine, Nicotine, N-Oleoylethanolamine, and O-Methyl-3-Epischelhammericine B) were primarily enriched in the epidermal cells and vascular bundles, displaying a similar dynamic pattern to that of organic acids, with an initial decrease, followed by an increase (Fig. 6a, b and Figure S5). This synchronized change may reflect the consumption of defensive resources during the early stages of pathogen invasion, followed by metabolic compensation and the establishment of resistance in later stages. In addition, a small number of amino acids and their derivatives were also enriched in the epidermis and vascular bundles. Amino acids not only serve as signaling molecules in plant stress responses but also enhance plant tolerance to biotic and abiotic stresses by regulating defense responses mediated by phytohormones such as JA and SA (Fig. 6a, b and Figure S5). Pathogens may exploit host amino acids as nutrient sources for their own metabolism, potentially leading to a local decrease in amino acid concentrations, which could explain the observed reduction in amino acid levels during the early stages of infection. As the infection progresses, plants may activate the expression of defense-related genes, upregulate the phenylpropanoid metabolic pathway (eg the conversion of phenylalanine into lignin and flavonoids), and the indole metabolic pathway, thereby promoting the synthesis of amino acid derivatives. The increase in amino acid derivatives in later stages may contribute to enhanced plant resistance.

Given that nicotine was the hallmark and most abundant alkaloid in tobacco, and represented a major defense-associated metabolite of particular agronomic and biological relevance, we prioritized it for functional validation of the observed alkaloid response. Accordingly, we evaluated a low-nicotine mutant line (*bbl*-KO) under pathogen challenge. The *bbl*-KO mutants developed more severe disease symptoms and accumulated higher bacterial loads than wild-type controls, supporting an essential defensive contribution of alkaloids in tobacco resistance. Consistently, exogenous nicotine application (Nicotine+) to wild-type plants markedly alleviated disease symptoms and suppressed bacterial proliferation relative to mock-treated controls. This indicates that nicotine alone can enhance resistance and partially compensate for reduced endogenous alkaloid-mediated defense during *P. syringae* infection (Fig. 6c to e). These results indicate that *P. syringae* infection elicits a coordinated, cell type-compartmentalized defense metabolic program, characterized by dynamic remodeling of epidermis- and vascular-enriched organic acids, alkaloids, and amino acid-related metabolism. Moreover, functional assays targeting nicotine strongly support that the alkaloid arm of this program contributes directly to restricting bacterial proliferation and alleviating disease progression in tobacco.

## Discussion

The interaction between pathogens and hosts involves complex metabolic signaling networks and immune regulatory cascades (Miao et al. 2025). By employing MALDI-MSI, we have constructed high-spatial-resolution metabolic maps of tobacco during *P. syringae* infection, enabling the spatial visualization of amino acids, sugars, organic acids, secondary metabolites, and defense hormones across different infection stages. This discovery identified critical transition points in metabolic reprogramming, providing a novel spatiotemporal framework for understanding the mechanisms underlying plant-pathogen interactions and establishing a methodological foundation for the precise identification of metabolic engineering targets in crop disease resistance breeding. Compared with previous static analyses, the combination of temporal sampling and spatial metabolomics significantly improves the resolution with which dynamic metabolic processes can be studied. While previous studies, such as spatial metabolomics of rice seeds revealing compartment-specific distributions of sugars, amino acids, and flavonoids, and integrative transcriptomic–metabolomic analysis of melon resistance uncovering coordinated suppression of flavonoids and induction of lignin biosynthesis (Ling et al. 2023; Zhao et al. 2023), have provided important insights into plant metabolism during development or stress, our study uniquely integrates spatial and temporal dimensions in a plant–pathogen system. This integration establishes a dynamic spatiotemporal framework for understanding defense responses. Future studies could integrate single-cell transcriptomics and spatial transcriptomic analyses to further elucidate the regulatory networks of functional genes within key metabolic hotspots. Furthermore, our findings provide a spatiotemporal characterization of defense-related phytohormones and secondary metabolites in tobacco across distinct infection stages, offering a framework for exploring the multilayered signaling regulation triggered by pathogen invasion.

Despite significant breakthroughs in the discovery of plant disease-resistant metabolites and the characterization of bioactive compounds, their spatial distribution remains largely unexplored. Unlike well-established methods for visualizing morphological structures and localizing macromolecules, accurately determining the spatial distribution of individual metabolites using optical techniques presents a significant challenge, particularly when most compounds are uncharacterized or unknown. Consequently, precise mapping of the specific sites of production, transport, and accumulation of many metabolites within tissues has remained elusive. As a recently developed tool, MALDI-MSI has been increasingly employed to explore the spatial distribution patterns of compounds within plant tissues (Bien et al. 2022). By leveraging high-resolution spatiotemporal data, our research delineates quantitative spatial patterns of multiple defense-associated metabolites, thereby filling a critical knowledge gap. The mesophyll cells serve as the primary site of photosynthesis, providing the energy and precursors for both primary and secondary metabolite biosynthesis (Allahverdiyeva et al. 2015). This study maps the spatial distribution patterns of these metabolites in tobacco leaves during *P. syringae* infection, offering important insights into the factors that influence their abundance, heterogeneous accumulation, and roles in tobacco defense against pathogens. Specifically, we found that alkaloids and organic acids are primarily enriched in the epidermis and vascular cells of tobacco leaves. Although the precise roles of alkaloids and organic acids in tobacco disease resistance remain uncertain, the spatial distribution patterns revealed here offer valuable insights into how biotic stresses influence these metabolites. These patterns shed light on their heterogeneous accumulation and their functions in tobacco's defense against pathogens. For instance, nicotine, the most representative alkaloid in tobacco, is known to effectively deter herbivorous insects and pathogenic microorganisms, contributing to tobacco’s resilience in challenging environments (He et al. 2024). Beyond tobacco, accumulating evidence from other plant systems supports a conserved defensive role of alkaloids in plant–microbe interactions. For example, Amaryllidaceae alkaloids in *Lycoris radiata* exhibit direct antimicrobial activity and shape the composition of plant-associated microbiota by selectively suppressing sensitive pathogens, while microbe-mediated enhancement of alkaloid accumulation further reinforces host resistance (Zhou et al. 2024). Similarly, in tea plants, caffeine functions as a jasmonate-induced defensive alkaloid whose biosynthesis is transcriptionally activated by the JA–MYC2–MYB regulatory module to enhance resistance against fungal pathogens, while being antagonistically modulated by gibberellin signaling to balance growth–defense trade-offs (Ye et al. 2025). Together, these studies support the view that alkaloids act as dynamic, hormone-regulated chemical defenses that integrate pathogen pressure with developmental constraints, consistent with the spatial and temporal patterns of alkaloid accumulation observed in our study. Spatial metabolomics results indicate that alkaloids like nicotine are primarily distributed in the vascular and epidermal cells, with levels increasing within 24 h of pathogen infection. Over time, the plant may adjust its resource allocation to avoid excessive depletion, or the threat from the pathogen may have weakened, leading to a decrease in the defense response. Organic acids play a dual role in plant physiology. They are essential intermediates in cellular respiration, participating in the tricarboxylic acid cycle to provide energy. Additionally, compounds like oxalic acid and SA inhibit fungal and bacterial growth, contributing to pathogen resistance (Wen et al. 2020; Lee et al. 2021). The spatial distribution of most organic acids mirrors that of alkaloids, with both responding within 24 h of pathogen infection and subsequently declining. As 2 important classes of secondary metabolites, organic acids and alkaloids exhibit complex and multilayered interactions within biochemical networks. SA triggers systemic acquired resistance and upregulates alkaloid biosynthesis genes, such as *PMT* for nicotine synthesis in tobacco, forming a dual barrier of chemical defense and immune signaling. Furthermore, organic acids acidify the pathogen infection sites, disrupting their membrane stability and enhancing the permeability and toxicity of alkaloids, including solanaceous tropane alkaloids. This synergistic action results in enhanced inhibition of pathogen growth (Ziegler and Facchini 2008; Maeda and Dudareva 2012). In addition, through meticulous spatiotemporal monitoring, this study tries to identify the key regulatory nodes in metabolite responses, providing evidence for the coordination of defense metabolic networks during pathogen invasion.

As the first physical and chemical barrier against pathogen invasion, plant epidermal cells secrete various defensive substances, including phenolic compounds, terpenoid secondary metabolites, and protease inhibitors. These compounds directly disrupt the pathogen's growth cycle and mitigate its toxic effects (Bidhendi et al. 2023). From a signal transduction perspective, epidermal cells play a dual role in plant immunity. They not only coordinate local and systemic immune responses through calcium ion oscillations in the cells surrounding the stomata, but also utilize membrane-localized pattern recognition receptors to specifically recognize pathogen-associated molecular patterns. This recognition activates the mitogen-activated protein kinase (MAPK) signaling cascade, promotes the biosynthesis of defense hormones such as SA and JA, and initiates NPR1-mediated systemic acquired resistance gene expression program (Wang et al. 2020; Zhang et al. 2021). It is noteworthy that the spatial metabolomics results of this study reveal that the accumulation levels of defensive secondary metabolites in the epidermal cells are significantly lower than anticipated. This observation may be closely related to the temporal dynamics of plant immune responses. During the initial stages of pathogen contact (0 to 6 hpi), epidermal cells likely prioritize the rapid synthesis of defensive compounds to establish an immediate protective barrier. As the infection progresses (>6 hpi), these metabolites may be actively transported or sequestered into vacuoles, resulting in a reduced steady-state concentration within the epidermal tissue. Furthermore, the sampling time points, which may not fully capture the early response phase, could also affect the observed spatial distribution of metabolites. However, we currently lack direct evidence (eg transporter gene expression profiles or metabolite quantification in phloem tissues) to confirm this mechanism. Therefore, we present it as a potential but unverified hypothesis, which warrants targeted investigation in future studies through integrative spatial metabolomics and transcriptomics approaches.

Within the intricate metabolic regulatory network, numerous metabolites exhibit close interactions with plant hormones. Studies have demonstrated that the antagonistic relationship between SA and JA in signal transduction plays a pivotal role in regulating nicotine biosynthesis in tobacco. Specifically, SA and JA are classically considered to act in an antagonistic manner during plant immune responses, with pathogen-induced SA signaling suppressing JA-mediated defense pathways (Rayapuram and Baldwin 2007). This antagonistic interaction provides a fundamental framework for understanding how plants balance distinct defense strategies. However, accumulating evidence suggests that effective resistance often relies not on a simple on–off switch between JA and SA pathways, but on their coordinated and context-dependent regulation. Consistent with this context-dependent model, targeted LC–MS/MS quantification revealed concurrent induction of SA and JA at 12 hpi, with distinct peak times (JA at 24 hpi and SA at 60 hpi) but sustained elevation of both hormones throughout infection relative to 0 hpi (Figure S6a). Comparative transcriptomic and phytohormone analyses between resistant and susceptible tea cultivars revealed that sustained and coordinated activation of JA and SA signaling, together with enhanced secondary metabolism, underpins effective resistance to *Apolygus lucorum* infestation (Ling et al. 2023). At the mechanistic level, recent genetic and molecular studies have further elucidated how this hormonal balance is dynamically tuned. Heterotrimeric G proteins were shown to fine-tune the antagonistic interplay between JA and SA signaling by stabilizing TCP14–JAZ regulatory complexes, thereby biasing immune responses toward SA-dependent defense during pathogen attack (Jia et al. 2025). Together, these findings highlight that JA–SA interactions operate through multilayered regulatory mechanisms, integrating signaling antagonism with precise molecular control to optimize plant defense outcomes. Moreover, the interaction between phenolic compounds and auxins activates a broader array of amino acids and their derivatives associated with pathogen resistance, thereby enhancing the plant's overall immune capacity (Li et al. 2023). Our findings reveal a positive correlation between the dynamics of phenolics, auxins, amino acids, and their derivatives, further substantiating this conclusion. Notably, ester metabolites also play a role in modulating auxin response in plants following pathogen infection. For instance, N-acyl homoserine lactones (AHLs) have been shown to activate auxin response promoters and upregulate auxin-related genes, thereby affecting plant growth and defense mechanisms (von Rad et al. 2008). Additionally, AHLs can enhance the plant's immune defense by elevating SA levels, particularly facilitating stomatal closure after *P. syringae* infection, thereby reducing pathogen invasion (Schenk et al. 2014). Phosphatidic acid, as a precursor for lipid synthesis, interacts with phosphatases in the ABA signaling pathway, preventing their translocation to the nucleus. This action alleviates the suppression of ABA, promoting stomatal closure and weakening pathogen invasion (Zhang et al. 2004). Finally, reactive oxygen species (ROS) and phosphatidic acid, as critical secondary messengers in the plant immune system, regulate ROS bursts induced by immune elicitors. This modulation of ROS levels following pathogen invasion enhances tobacco's antibacterial and antifungal activities (Qi et al. 2024). These insights hold significant potential for crop disease resistance breeding and provide a theoretical foundation for developing strategies to enhance plant immunity through metabolic regulation (Figure S6b).

This study comprehensively investigated the spatiotemporal metabolic reprogramming in tobacco leaves during *P. syringae* infection using a combination of physiological observations and spatial metabolomics powered by MALDI-MSI. Infection caused progressive and stage-dependent phenotypic and cytological changes, including dynamic stomatal opening/closure and mesophyll structural remodeling, providing a physiological framework for subsequent metabolic shifts. A total of 1,399 MSI features spanning 14 metabolite categories were annotated, revealing broad metabolic remodeling during infection, with organic acids, amino acids and their derivatives, and alkaloids representing major metabolite classes. Temporal analysis highlighted a staged metabolic response. Early infection (12 hpi) was characterized by the upregulation of organic acids and terpenoids, potentially serving as initial defense barriers or signaling molecules. This was followed by a pronounced accumulation of alkaloids by 60 hpi, underscoring their critical role in later-stage defense. Flavonoids and terpenoids exhibited a bidirectional regulatory trend, suggesting a dynamic allocation of defense resources. MALDI-MSI enabled direct visualization of dynamic metabolic remodeling, revealing distinct spatial patterns of metabolite distribution. ROI-based spatial annotation and quantification, guided by leaf anatomical features and MSI-derived spatial distribution patterns, identified anatomically aligned metabolic compartments, with key defense metabolites exhibiting cell-type-specific preferences and directional redistribution. Specifically, epidermal cells emerged as a critical hub for the accumulation of defense-associated organic acids, alkaloids (including nicotine), and amino acid derivatives, which showed dynamic changes indicative of consumption during early invasion and compensatory synthesis later in infection. Vascular bundles primarily concentrated defense hormones like SA and JA, and certain auxin derivatives, suggesting their crucial role in systemic signaling and defense. Functional validation experiments provided robust evidence for the roles of key metabolites and hormones. Exogenous application of identified defense compounds, such as calystegine C1, L-phenylalanine, artemisinin, defense hormones like JA and SA, and defense alkaloids like nicotine, significantly enhanced tobacco resistance, reduced lesion development, and suppressed bacterial proliferation. Genetic studies on JA and nicotine biosynthesis further reinforced the importance of these pathways in restricting pathogen growth. Collectively, this work establishes a spatiotemporal metabolic atlas of tobacco wildfire disease progression and demonstrates that effective resistance emerges from coordinated, tissue-compartmentalized deployment of defense metabolites and hormone-linked pathways, providing a resource for identifying metabolic targets and pathways for resistance improvement.

## Supplementary Material

kiag623_Supplementary_Data

## Data Availability

All data generated or analyzed in this study are included in the materials and methods section of this article.
