# Supplementary material for: Spatiotemporal metabolomics of tobacco leaves in response to Pseudomonas syringae infection
Source: Plant Physiol. 2026 Aug 20;202(1):kiag623. doi: 10.1093/plphys/kiag623 (PMC13530300; doi:10.1093/plphys/kiag623)
Supplement: Supplementary file 1 [file kiag623_supplementary_data.zip › Supplementary Figures.docx]

**Figure S1.** Spatial disease progression and histological changes in tobacco leaves following *P. syringae* infection. (A) Representative images showing spatial disease symptoms in different regions of tobacco leaves across different time points after *P. syringae* inoculation. The 60 hpi infected leaf image is the same representative image shown in Fig 1A and is used here to indicate the sampling regions for bacterial quantification. Right panel shows quantitative analysis of *P. syringae* abundance in different leaf regions at 60 hpi. (B) Scanning electron micrographs of the adaxial leaf epidermis at 0, 12, 24, and 60 hours post-infection (hpi) with *P. syringae*, shown as low-magnification overviews (Scale bars = 100 µm) with high-magnification insets (Scale bars = 10 µm) of individual stomata. (C) Quantitative analysis of stomatal traits at the same time points: left, Stomatal length (μm); middle, Stomatal width (μm); right, Stomatal opening percentage. Bars represent mean ± SD (n = 3). Different letters indicate significant differences among time points (P ≤ 0.05, one-way ANOVA with Tukey’s HSD).

**Figure S2.** Stage-dependent differential metabolite dynamics revealed by Mfuzz temporal trajectories. (A) Mfuzz clustering of metabolites based on their temporal trajectories across 0, 12, 24, and 60 h, showing representative time-dependent patterns. (B) Venn diagram showing differentially expressed metabolites (DEMs) identified from Mfuzz Clusters 3 and 4 at 12, 24, and 60 hpi relative to 0 hpi, together with statistical classification of the DEMs shared across all time points by metabolite class. (C) Heatmap showing the correlation patterns of the shared DEMs from Mfuzz Clusters 3 and 4 (excluding the “Others” class) with vascular, mesophyll, and epidermal regions.

**Figure S3.** Differentially expressed metabolite analysis during *P. syringae* infection. (A) Venn diagram of upregulated and downregulated metabolites. (B) Statistical analysis of upregulated, downregulated, and non-significantly changed metabolites for each class at different time points.

**Figure S4.** Time-resolved metabolite changes and *LOX* overexpression–mediated resistance to *P. syringae* in tobacco. (A) The relative abundance of various hormones in tobacco during *P. syringae* infection. Significant differences are assessed using one-way analysis of variance (ANOVA) with Tukey's HSD test. (B) Representative disease phenotype of wild-type control (CK) and LOX-overexpression lines (*LOX3*-OE, *LOX4*-OE, *LOX6*-OE) at 0, 12, 24, and 60 hpi. The same representative wild-type CK images were used as the shared CK control in Fig 5C, Fig 6C, and Fig S4B. (C) Quantification of relative lesion area in CK and *LOX*-overexpression plants at the indicated time points. (D) Quantification of *P. syringae* biomass in leaves of CK and *LOX*-overexpression plants at 0, 12, 24, and 60 hpi (log10 ng pathogen DNA per 100 mg leaf tissue).

**Figure S5.** The relative abundance of various alkaloids, organic acids, amino acids, and their derivatives in tobacco during *P. syringae* infection.

**Figure S6.** Coordinated SA/JA dynamics and a proposed metabolite–hormone interaction model in infected tobacco leaves. (A) Time-course quantification of jasmonic acid (JA) and salicylic acid (SA) levels in infected tobacco leaves at 0, 12, 24, and 60 hpi. Hormone concentrations were determined using targeted LC-MS/MS with authentic standards. (B) Dynamic response model of key metabolites during *P. syringae* infection in tobacco leaves. For multiple-group comparisons in panel A, one-way ANOVA followed by the Tukey–Kramer test was used; different lowercase letters above bars indicate significant differences at *P* < 0.05.
